# The (mis)use of fetal viability as the determinant of non-criminal abortion in the Netherlands and England and Wales

**DOI:** 10.1093/medlaw/fwad015

**Published:** 2023-05-30

**Authors:** Samantha Halliday, Elizabeth Chloe Romanis, Lien de Proost, E Joanne Verweij

**Affiliations:** Centre for Ethics and Law in the Life Sciences, Durham Law School, Durham DH1 3LE, UK; Centre for Ethics and Law in the Life Sciences, Durham Law School, Durham DH1 3LE, UK; Edmond and Lily Safra Center for Ethics and Petrie-Flom Center for Health Law Policy, Biotechnology and Bioethics, Harvard University, Cambridge, Massachusetts 02138, United States; Department of Obstetrics and Gynecology, Erasmus MC, The Netherlands; Department of Medical Ethics, Philosophy and History of Medicine, Erasmus MC, The Netherlands; Department of Neonatology, Erasmus MC, The Netherlands; Department of Obstetrics and Gynecology, Erasmus MC, The Netherlands; Department of Obstetrics, Leiden University Medical Center, The Netherlands

**Keywords:** Abortion, Criminal law, Fetal viability, Medicalization, Neonatal viability, Viability

## Abstract

Time plays a fundamental role in abortion regulation. In this article, we compare the regulatory frameworks in England and Wales and the Netherlands as examples of the centrality accorded to viability in the determination of the parameters of non-criminal abortion, demonstrating that the use of viability as a threshold renders the law uncertain. We assess the role played by the concept of viability, analysing its impact upon the continued criminalization of abortion and categorization of abortion as a medical matter, rather than a reproductive choice. We conclude that viability is misconceived in its application to abortion and that neonatal viability (relating to treatment of the premature infant) and fetal viability (related to the capacity to survive birth) must be distinguished to better reflect the social context within which the law and practice of abortion operate. We show how viability thresholds endanger pregnant people.

## I. INTRODUCTION

Time has a fundamental role in the regulation of abortion. It is used to draw lines delineating the boundaries of abortion, setting the dividing line between lawful and unlawful abortions.^1^ The point in gestation at which those lines are drawn varies between jurisdictions, but in many jurisdictions the legal bright line adopted for that boundary is *viability*, generally understood as the point at which the fetus could survive, albeit with medical assistance, outside the uterus.^2^ A large body of philosophical literature critiques the moral relevance of viability as the point at which the fetus attains significance and should be protected.^3^ However, there is much less literature that critiques the problems of enshrining a viability threshold in the law.^4^ The focus of this article is upon the use of viability as a legal construct and the uncertainty that this creates.

Viability as a concept was initially developed to determine the boundaries of acceptable treatment for extremely premature neonates,^5^ a very different context to the termination of pregnancy. Nevertheless, since the mid-twentieth century, viability has become a central feature in abortion regulation, becoming a threshold beyond which a fetus can no longer be legally aborted, or when permissible grounds for a termination of pregnancy are severely restricted. However, viability is a difficult concept on which to base a law, or even professional guidance. The concept is ambiguous, complex, and difficult to apply in practice. It is a moveable threshold, determined by fetus-specific and external considerations, rather than a universal standard. Viability is innately fetus-specific, dependent upon a number of intersecting fetal characteristics such as sex and weight.^6^ Furthermore, fetal outcomes cannot be divorced from external considerations like geography and, often relatedly, resources.^7^ When discussed as a standard, viability is based on the ‘human interpretation of statistical probabilities’ applied to fetuses as a group without consideration of the contextual factors influencing the likelihood that any specific fetus could or would survive.^8^ This makes viability as a concept ill-suited to laws determining the scope of criminal liability, where a person’s access to healthcare and a doctor’s liberty and licence to practice medicine are at stake. Moreover, the import of a medical concept designed to determine the appropriateness of treatment intended to preserve life in the case of extremely premature birth into the context of abortion, where, by its very nature, the fetus is not intended to survive, is a conceptually illegitimate ground on which to base abortion regulation. The two contexts are entirely distinct.

The World Health Organization (WHO) specifically recommends ‘against laws and other regulations that prohibit abortion based on gestational age limits’ because such laws delay access to abortion (especially at later gestations) and are associated with higher incidences of unsafe abortion and maternal morbidity and mortality.^9^ Despite the inherent uncertainty of gestational age limits, viability is often adopted as a threshold concept in laws regulating abortion. The regulatory models adopted in England and Wales and the Netherlands provide two particularly good examples of the centrality accorded to viability in the determination of the parameters of non-criminal abortion, restricting post-viability abortion to cases where the person’s life or health is at risk, or where the termination of pregnancy is based upon fetal anomaly (embryopathic) grounds. In both jurisdictions, there is significant concern that advances in perinatal medicine that enable neonates born at earlier gestations to survive might lead to a reduction in the time limit for abortion.^10^ Our contribution to the literature is particularly timely in the light of the current review of the Dutch guideline on perinatal care in case of extremely premature birth.^11^ The likely revision of the perinatal guidance, to recognize that preterm neonates can survive *prior* to the 24th week of gestation, could lead to the lowering of the upper limit for lawful abortion.^12^ This possibility thus necessitates an examination of the use of fetal viability to denote the boundaries of lawful abortion and the potential adoption of a single threshold for abortion *and* the care of premature neonates.

This article compares the regulation of abortion in England and Wales and the Netherlands, evaluating the role played by fetal viability in the law. While there are significant differences in the law relating to abortion in doctrinal terms, there is much commonality in the practice of abortion. Both jurisdictions are often viewed as examples of places with ‘liberal abortion laws’ in Europe.^13^ This is a misconception. In both jurisdictions, the viability threshold acts as a significant barrier to accessing abortion services. Even before viability, access is limited by being contingent upon medical discretion, with doctors being placed in the position of gatekeepers to lawful abortion.^14^*After* viability, access is much more restricted, with abortion only being available on the basis of a ‘maternal health/life’, or embryopathic indication. While we make our arguments with specific reference to these jurisdictions, our conclusions about the impetus for the decriminalization of abortion and/or removal of gestational age limits from abortion regulation have broader applicability and provide support to calls from international actors, such as the WHO^15^ and United Nations Special Rapporteurs,^16^ for decriminalization.

We begin the next Section II by considering the legal frameworks regulating abortion in England and Wales and the Netherlands. In Section III, we compare the legal frameworks and the role played by the concept of viability therein, analysing its impact upon the continued criminalization of abortion and the categorization of abortion as a medical matter, rather than the exercise of an individual’s autonomy. Finally, we argue that the concept of viability is misconceived in its application to abortion and that neonatal viability (relating to treatment of the premature infant) and fetal viability (related to the capacity to survive birth) must be distinguished to ensure better access to late-term abortion for those who need it (in Section IV). Moreover, we demonstrate that the use of viability as a threshold renders the law uncertain and that, by embedding this concept in the regulatory framework, the law fails to reflect the social context within which the law and practice of abortion operates.

## II. LEGAL FRAMEWORKS

### A. England and Wales

Abortion is a crime in England and Wales. Section 58 of the Offences Against the Person Act 1861 (OAPA) stipulates that any person who procures a pregnant person’s miscarriage ‘by any means whatsoever’ with the intent of procuring miscarriage is guilty of a criminal offence.^17^ This offence can be committed throughout pregnancy^18^ and carries a maximum sentence of life imprisonment. In addition, section 1 of the Infant Life (Preservation) Act 1929 (ILPA) criminalizes ‘child destruction’, committed when a person ‘with intent to destroy the life of a child capable of being born alive, by any wilful act causes a child to die before it has an existence independent of its mother’. The term capable of being born alive is not defined further in the 1929 Act, although it is noted that a fetus is to be *presumed* ‘capable of being born alive’ from 28 weeks’ gestation.^19^ Unlike the ‘unlawful procurement of miscarriage’ therefore, ‘child destruction’ can only be committed *after* a fetus is ‘viable’ later in a pregnancy.^20^

#### 1. Grounds for abortion

The Abortion Act 1967 (AA) specifies the conditions in which *doctors* will not be guilty of either of these criminal offences when performing or supervising termination of pregnancy. Termination of pregnancy is lawful where two doctors have formed the opinion, in good faith, that:

- the pregnancy has not exceeded 24 weeks and continuing the pregnancy is a greater risk than termination to either the pregnant person’s physical or mental health or to any existing children of their family; or
- termination is necessary to prevent ‘grave permanent injury’ to the pregnant person’s physical or mental health; or
- continuance of the pregnancy poses a greater risk to the pregnant person’s life than termination; or
- there is a ‘substantial risk’ that if the fetus is born it would ‘suffer from such physical or mental abnormalities as to be seriously handicapped’. ^21^

When making a determination about a pregnant person’s physical or mental health, doctors should take into account their ‘actual and reasonably foreseeable circumstances’.^22^ Consequently, the first ground—often referred to as the ‘social ground’ for abortion^23^—is read incredibly broadly, and in practical terms renders every early pregnancy legally terminable.^24^ The risks of abortion (medical or surgical) early in a pregnancy will always be lesser than those associated with carrying a pregnancy to full-term and childbirth,^25^ particularly before 13 weeks' gestation. It is often on this basis that the 1967 Act is praised for rendering abortion easily accessible.^26^ Having said that, the Act does not enable ‘abortion on demand’,^27^ or on ‘social grounds’. Although often described as a social indication, section 1(1)(a) is, in its construction, a socio-*medical* indication requiring the abortion to be justified *in clinical terms*, albeit considering a person’s social context and broader welfare.^28^

#### 2. The centrality of viability

The AA 1967 does not make any explicit reference to fetal viability, but it does instil a threshold *before* which point access is *easier*, and *after* which point it is *more difficult*. Before 24 weeks, section 1(1)(a) permits the socio-medical abortions described above. After 24 weeks, some proof of a danger to the pregnant person or the fetus is required.^29^ This is harder to establish.^30^ The 24-week threshold is not described in the Act itself as being the identified point *because* it reflects the point at which a fetus might be deemed ‘viable’, but it correlates with the point in gestation at which viability is most often referenced,^31^ and so instils ‘an implicit viability threshold’.^32^

The offence of ‘child destruction’ was introduced, via the 1929 Act, to fill the gap between abortion and homicide (which requires live birth) where a fetus is killed during birth.^33^ The victim in this situation is recognized as a fetus ‘capable of being born alive’^34^ and thus defined *directly by comparison to a newborn*. As we have noted above, there is a statutory presumption that at 28 weeks’ gestation the fetus will constitute a ‘child capable of being born alive’ and this makes it clear that the offence of child destruction is *not* limited to the fetus killed during birth. ‘Capable of being born alive’ can be interpreted in different ways, raising the question whether the capacity for live birth suffices, or is the capacity for survival required? It has been argued that the explicit viability threshold in English law—codified in the offence of child destruction—means ‘capable of being born alive and surviving for a time by breathing, rather than being born alive and surviving in the longer term’.^35^ The purpose of the presumption is to relieve the prosecution of the need to prove that a fetus meets the criterion of being a child capable of being born alive from the 28th week of pregnancy onwards.^36^

Although prosecutions for child destruction generally relate to individuals who have assaulted the pregnant person (rather than the pregnant person themselves, or a doctor),^37^ it has been recognized in case law that the offence might also be committed when a later-term abortion is conducted.^38^ Few prosecutions of child destruction are brought every year, still fewer convictions are achieved due to the need to demonstrate intent to destroy life.^39^ However, the convictions have all related to the destruction of life *after* 28 weeks’ gestation.^40^ Nevertheless, the presumption does not preclude a finding that a fetus or fetuses earlier in gestation can have the capacity to be born alive^41^ and it is suggested that the 24-week time limit applied to socio-medical abortions lends support to the view that child destruction may be committed from *at least* that point onwards.

#### 3. Post-viability abortion

After 24 weeks’ gestation, access to abortion becomes much harder, requiring either a maternal indication or fetal anomaly (under sections 1(1)(b)–(d) of the AA 1967).

##### a. Maternal indication

Later in pregnancy, there is a defence to both child destruction (under the 1929 Act) and unlawful miscarriage (under the 1861 Act) where there is a risk to life, or a risk of grave, permanent injury to the pregnant person’s physical or mental health. Section 1(1)(c) establishes a comparative standard, requiring the risk to the person’s life be greater if the pregnancy were to continue than if it were terminated. There is no such stipulation where the abortion is *necessary* to prevent ‘grave and permanent damage to health’,^42^ but the threshold of risk is high and is limited to serious conditions, such as those that might lead to kidney, brain or heart damage.^43^ These provisions are broad; terms like ‘grave’ and ‘permanent’ are undefined, to ensure that wide discretion is conferred upon clinicians. Furthermore, the requirement is that doctors form their opinion that the indication is satisfied in good faith, rather than that their opinion is correct and therefore, in practice, doctors are able to intervene to end later-term pregnancies to preserve a pregnant person’s life or health without fear of prosecution.

##### b. Fetal anomaly

There is no time limit for abortion where doctors, forming their opinion in good faith, believe that there is a ‘substantial risk’ that the fetus will be ‘seriously handicapped’.^44^ Neither of these terms, however, are defined by statute or in case law, with the lacuna being left to be filled by professional guidance.^45^ This ground has attracted considerable criticism for being too broad^46^ and has resulted in judicial review challenges to decisions to allow abortion for conditions such as Down’s syndrome^47^ and cleft palate^48^ later in pregnancy. Clinical guidance from the Royal College of Obstetricians and Gynaecologists (RCOG) states that a serious handicap will be a non-trivial condition that is not readily correctable and causes significant suffering or inability to participate in society.^49^ The RCOG working party guidance indicates that whether a risk is substantial is not merely a statistical calculation, but depends ‘upon factors such as the nature and severity of the condition and the timing of diagnosis, as well as the likelihood of the event occurring’.^50^ This suggests that even a moderate risk of a severe condition may satisfy the criterion. Doctors are advised to seek advice from specialists to demonstrate that they formed their opinion about the risk or the severity of the fetal anomaly in good faith.^51^ Notwithstanding the broad discretion afforded to clinicians, the challenges to clinical judgement evident in cases like *Jepson* and *Crowter* have left some doctors feeling vulnerable to prosecution, resulting in a preference to perform embryopathic abortions prior to the expiry of the 24 week time limit applied to section 1(1)(a) AA 1967.^52^

### B. The Netherlands

Abortion is regulated in the Netherlands by the Dutch Penal Code (WvS),^53^ the Termination of Pregnancy Act 1981 (Wafz),^54^ and the Termination of Pregnancy Decree 1984 (Bafz).^55^ Situated directly after the section of the Penal Code relating to crimes against life, Article 296(1) of the WvS stipulates that:

Any person who gives a [person] treatment, when he knows or has reasonable cause to suspect that this treatment may terminate the pregnancy, shall be liable to a term of imprisonment not exceeding four years and six months or a category four fine.

Setting out an exception to this provision, Article 296 (5) of the WvS provides that a termination of pregnancy will not be punishable if it is performed by a doctor in a licensed hospital, or clinic, in accordance with the Wafz.

The Explanatory Memorandum to the Act sets out the legislation’s three broad aims: (i) to provide assistance to pregnant persons in an emergency situation because of an unwanted pregnancy; (ii) to protect unborn life; and (iii) to safeguard pregnant persons’ health, both in relation to the performance of the termination itself and through good aftercare.^56^ Each aim is considered in turn below. As these aims make clear, the regulatory model adopted in the Netherlands emphasizes that the fetus is morally significant, albeit not a legal person and underscores the exceptionalism that characterizes abortion regulation—abortion is framed as neither a purely medical matter to be left to doctors to determine, nor simply a matter of choice for pregnant people.

#### 1. Assisting pregnant persons in an emergency situation because of unwanted pregnancy

In the same way that the AA 1967 excludes criminal liability under the OAPA 1861 and the ILPA 1929, the Wafz excludes liability under Article 296 of the WvS, but it does so in broad terms, excluding liability where the pregnant person is in a state of emergency and the termination is performed in accordance with the requirements set out in the Act. As was common in legislation during the later part of the twentieth century, the Dutch law underlines the exceptionalism attributed to abortion, categorizing abortion as a measure to assist pregnant persons in an emergency situation as a result of an unwanted pregnancy, rather than recognizing a right to termination.

The Act does not attempt to define what will constitute an emergency, adopting a general proposition and leaving the determination of whether the pregnant person is in a state of emergency to be determined by the individual and their doctor.^57^ In practice, this provides wide access to abortion in the Netherlands as any unwanted pregnancy can be regarded as constituting an emergency situation for the individual. Nevertheless, it is unfortunate that the second review of the Wafz did not recommend that the Act be amended to recognize autonomy as the justification for abortion, rather than a ‘state of emergency’ as assessed by a third party. Moreover, as discussed below, the general state of emergency will only permit abortions performed *prior* to viability; thereafter, the individual’s state of emergency as a result of unwanted pregnancy becomes irrelevant and protection of fetal life is prioritized absent a maternal or embryopathic indication.

Unlike the AA 1967, the Wafz does not set out any indications that will justify an abortion, relying solely upon the pregnant person being in a state of emergency based upon an unwanted pregnancy. Neither medically indicated nor embryopathic abortions are provided for by the Wafz,^58^ instead they are subject to the general prohibition of abortion,^59^ leaving the doctor dependent upon the justification of necessity to escape criminal liability. As we now demonstrate, viability plays an essential role in these contexts.

#### 2. Protecting unborn life—the significance of viability

The second aim set out in the Explanatory Memorandum is the protection of unborn life. By retaining the crime of abortion in the Penal Code,^60^ albeit with an exemption from criminal liability for doctors performing an abortion to resolve a pregnant person’s state of emergency in accordance with the Wafz,^61^ the legislature expressed the exceptionalism of abortion. Abortion is framed not merely as a medical procedure subject to the general law and professional guidance relating to medical treatment, but as an area of healthcare requiring additional regulation. The Wafz seeks to protect unborn life through a combination of substantive and procedural rules (the due care criteria) designed to ensure careful decision-making. It requires that:

if the [person] considers that [their] emergency situation cannot be terminated in any other way, the doctor shall satisfy himself that the [person] made and maintained [their] request voluntarily, after careful consideration and in awareness of [their] responsibility for unborn life and of the consequences for [their]self and others.^62^

The explicit reference to the pregnant person’s responsibility for unborn life underlines the expectation that they should continue a pregnancy. Moreover, although there is stress laid upon abortion being the individual’s decision, the Act requires the doctor to be satisfied that there is no other way to resolve the pregnant person’s emergency situation than to terminate the pregnancy.^63^ To this end, the doctor is required to discuss alternatives with the pregnant person,^64^ and a mandatory reflection period is set out designed to ensure a well-considered decision.^65^

No time limit for permissible abortions is set out in the Wafz. However, the Explanatory Memorandum specifically states that termination of pregnancy in the case of a viable fetus will remain a criminal offence and constitute an instance of intentional deprivation of life according to Article 82a of the WvS, an amendment to the Penal Code introduced by the Wafz.^66^ Therefore, the parameters of lawful abortion in the Netherlands are set by Article 82a of the WvS, which provides that ‘Taking the life of a person … shall include: the killing of a fetus that can reasonably be expected to have the ability to survive outside the mother’s body.’ If a fetus is born alive after a post-viability abortion, no offence will have been committed. However, if a fetus is stillborn, the abortion performed by a doctor will constitute a homicide,^67^ concurrently with abortion,^68^ as the exception set out in Article 296(5) of the WvS does not apply after viability.^69^ Applied in this manner, the legality of abortion provision is tied to the potential for the fetus’ survival after birth.

While the viability threshold is an external time limit, the provisions of the Wafz are construed in accordance with Article 82a of the WvS, thus excluding post-viability abortion from the scope of the Act. Notably, the provision does not specify when viability can reasonably be expected to occur, or even set up a statutory presumption of viability such as that found in the ILPA 1929. This is problematic as law, particularly criminal law, should be certain in scope and there are multiple definitions attributed to viability. Reflecting the scientific consensus of the time, the Explanatory Memorandum adopts 24 weeks as the viability threshold,^70^ a position confirmed in 1991 by the Supreme Court (*Hoge Raad*).^71^ The court identified 24 weeks as the upper limit for abortion, noting that medical science would support a reasonable expectation of viability from that point and that all Article 82a of the WvS requires is an expectation that the fetus would survive birth, *even if only for a short time*. Importantly, the Supreme Court stressed that, in practice, the upper limit for abortion would be significantly lower than 24 weeks, stating that a margin of inaccuracy of 4 weeks should be taken into account when dating a pregnancy, reduced to 2 weeks when more advanced diagnostic tools are used.^72^ This means that, applying a safety margin, the true threshold for abortion as a means of resolving the individual’s emergency situation is 20–22 weeks in the Netherlands.^73^

The impact of Article 82a of the WvS is to impose a flexible time-limit upon abortion, enabling viability (and thus the threshold for lawful abortion) to move alongside developments in perinatology. There have been significant developments in perinatology since the Wafz came into force in 1984, advances that have enabled premature children to survive at progressively earlier gestations.^74^ When the Wafz was enacted, extremely premature neonates born below 26 weeks’ gestation were not treated and so did not survive in the Netherlands.^75^ However, the current professional guidance issued by the Dutch Societies of Paediatricians (NVK) and for Obstetrics and Gynaecology (NVOG) states that treatment for extremely premature neonates should be offered to those born at 24 weeks’ gestation, after consultation with the parents.^76^ Thus, the current boundaries for lawful abortion and active treatment for premature neonates intersect at 24 weeks. The NVK and NVOG guideline is currently under review, giving rise to concern that if changes are made to determinations about the viability and the associated appropriateness of treatment in the neonatal intensive care setting, those changes will impact upon the gestational threshold for abortion and reduce the time limit for abortion. Addressing this concern, both the Second Review of the Wafz and the 2022 Late Termination of Pregnancy Evaluation report recommended that the link between the threshold for abortion and medical advances in the survival of extremely premature births should be severed and that the 24-week time limit currently applicable to abortion on non-medical grounds should be inserted into the Wafz.^77^ The new Dutch government has yet to take action on these recommendations.

#### 3. Safeguarding pregnant person’s health both in relation to the performance of the termination itself and through good aftercare

The Wafz and Bafz go further than the AA 1967, providing a detailed framework for the provision of abortion, emphasizing the need to safeguard pregnant persons’ health going beyond the requirement in the Abortion Act 1967 that abortion is performed only by a registered medical practitioner in licensed premises. The requirements of due care are intended to ensure that the pregnant person is able to make a careful decision regarding the termination of their pregnancy.^78^ Underscoring the narrative of pregnant persons as requiring guidance and being unable to make a responsible decision to terminate a pregnancy without assistance, requirements include that the attending doctor must inform the pregnant person about alternatives to abortion and that access be provided to psychology and social care professionals to assist them, should they so wish.^79^ Through this detailed framework,^80^ the distinctive nature of abortion compared to other medical procedures is highlighted, it is subjected to additional rules outside the general legal framework applicable to medical treatment, thereby stigmatizing both the pregnant person and the doctor.^81^

#### 4. Abortions falling outside the scope of the termination of pregnancy act

The Wafz is applicable only to abortions performed prior to viability and conceptualizes abortion as a solution for a pregnant person’s emergency situation caused by unwanted pregnancy. Thus, the Act is designed to regulate abortion of the type that would be covered by section 1(1)(a) AA 1967 in England and Wales. For that reason, the Wafz does not provide justifications for abortion in the case of a threat to the pregnant person’s life or health (maternal indication),^82^ or abortion based upon fetal anomalies (embryopathic indication),^83^ rendering post-viability ‘therapeutic’ abortion a crime.^84^

Abortions performed on maternal or embryopathic grounds prior to viability will result in the termination of what will often be very much wanted pregnancies; however, they will be permitted as cases of resolving the pregnant person’s emergency situation and benefit from the indemnity set out in Article 296(5) of the WvS. Once the fetus attains viability, pregnancy cannot be lawfully terminated under the Wafz. However, that is not to say that abortion is not available in such cases; rather, recourse is had to the justification of necessity^85^ and what Sjef Gevers has termed a restrained prosecution policy.^86^ A key distinction between pre- and post-viability abortion is that while abortion prior to 24 weeks is regarded as a matter for clinical expertise, the performance of ‘late’ abortions is subjected to scrutiny, highlighting the enhanced status of the fetus in law from the point at which viability will generally occur.

##### a. Fetal anomaly

In many cases, fetal anomalies are not identified prior to the 20-week anomaly scan, leaving little time for the pregnancy to be terminated prior to viability. The impact of Article 82a of the WvS is to render all abortions after viability a crime against life, concurrently with exposing the doctor to liability for terminating a pregnancy under Article 296 of the WvS. Nevertheless, a framework has been established to regulate abortion on the basis of fetal anomaly through the 2016 Ministerial Regulations of the Assessment Committee for Late Terminations of Pregnancy and Termination of Life in Neonates.^87^ Two categories of late termination of pregnancy for fetal anomaly are set out.^88^ Category 1 consists of cases where the fetus would be expected to die immediately after birth; for example, anencephaly, double pulmonary hypoplasia, trisomy 13 (Patau’s syndrome), and 18 (Edward’s syndrome). The second category of late terminations of pregnancy concerns cases where one or more conditions are present in the fetus which lead to serious and irreparable functional disorders; for example, spina bifida. Thus, abortion on embryopathic grounds is not limited to cases of fatal fetal anomaly, but the ground is considerably narrower than that set out in section 1(1)(d) of the AA and will not include late onset conditions, such as Huntington’s, or conditions perceived as less severe, such as Down’s syndrome. The impact of this is significant as in cases not falling within either category the termination can only lawfully take place prior to 24 weeks on the general emergency ground, leaving little time after diagnosis to decide about termination. This leads to a bottleneck in abortion provision, forcing some people to access later abortion by travelling to another country or resorting to purchasing abortion pills on the internet.^89^

In both categories, the termination must be reported to the municipal pathologist by the attending doctor as a case of unnatural death. Following a post-mortem, the municipal pathologist will notify the public prosecutor who has to give a declaration of no objection to burial, or cremation before either can take place. Moreover, the attending doctor is required to submit a report detailing compliance with the due care criteria to the Assessment Committee for Late Termination of Pregnancy and Termination of Life in Newborns. The committee (made up of four doctors, a lawyer and an ethicist) is responsible for assessing all reported cases of late termination of pregnancy (*both* category 1 and 2) *and* cases of termination of life in neonates, underscoring the increased status endowed upon the fetus after viability would generally be expected to occur.

In category 1 (fatal anomaly) cases, there is no reasonable expectation that the fetus will survive outside the uterus and therefore it is not considered viable. Article 82a of the WvS finds no application, but Article 296(5) of the WvS and the provisions of the Wafz do apply. Upon reviewing the case, if the Committee finds that the attending doctor acted in accordance with the Wafz and professional guidance, they will be deemed to have complied with the due care criteria,^90^ and will be exempt from criminal liability. The file will be closed. If, however, the Committee finds that the doctor has not complied with the due care criteria, it will inform the Health and Youth Care Inspectorate (IGJ) which may initiate disciplinary proceedings, or can report the case to the Board of Procurators General (BPG) if it believes that an offence has been committed.

Category 2 (conditions leading to serious and irreparable functional disorder) cases are more complex because the fetus is considered viable on the basis of gestational age, creating the potential for concurrent liability under Articles 289 (murder) with 82a, and 296 (abortion) of the WvS.^91^ Stricter scrutiny is therefore applied to termination for a non-fatal anomaly both in terms of the identification of the due care criteria to be applied and the institution of a double review after the termination. While the regulations define due care by reference to professional practice and guidelines in category 1 cases, specific due care criteria are applicable to category 2 cases.^92^ These cumulative criteria require that the attending doctor:

1. is convinced that the anomalies are such that medical intervention after birth would be futile according to prevailing medical opinion and there is no reasonable doubt about the diagnosis and the prognosis based on it;
2. is convinced that the fetus is currently suffering, or will foreseeably suffer, without prospect of improvement;
3. has fully informed the parents of the diagnosis and the prognosis, reaching the consensus that there is no reasonable alternative to termination;
4. the pregnant [person] has explicitly requested termination of the pregnancy due to [their] physical or psychological suffering caused by the situation;
5. the doctor has consulted at least one other, independent doctor, who has given [their]written opinion on the aforementioned due care requirements, or, if an independent doctor could not reasonably be consulted, has consulted the medical team, which has given its written opinion on the above;
6. the pregnancy was terminated with due medical care.^93^

The criteria are broadly drafted and the LTP Evaluation 2022 noted that doctors find it particularly difficult to interpret the requirement of hopeless and unbearable suffering,^94^ and to be certain that the ‘no reasonable doubt’ threshold is satisfied in the absence of DNA diagnosis, where the severity assessment can be made solely on the basis of imaging.^95^ Only 33% of respondents to the evaluation reported that they found the category 2 due care requirements sufficiently clearly formulated.^96^ Moreover, only 57% of respondents said that it is mostly clear which category cases fall into.^97^ However, the distinction is crucial because, unlike category 1 cases, the Committee *must* forward its finding in all category 2 cases to the Board of Procurators General,^98^ even if it concludes that the doctor complied with the due care criteria. While Committee findings carry significant weight in the decision of whether to prosecute doctors, prosecuting authorities are not bound by the findings of the Committee in deciding whether to prosecute the attending doctor.^99^ Category 2 cases thus will feel risky for doctors.

The scheme for reviewing and reporting instances of late termination of pregnancy based on an embryopathic indication resembles the scheme adopted by the Termination of Life on Request and Assisted Suicide (Review Procedures) Act 2001, but with an important distinction in category 2 cases. The automatic referral in these cases has a chilling effect upon doctors’ willingness to perform late abortions,^100^ and its inclusion is particularly surprising given that the automatic referral to the prosecuting authorities in assisted dying cases^101^ was abolished by the Termination of Life on Request and Assisted Suicide (Review Procedures) Act 2001 *precisely because of* its impact upon doctors’ willingness to report euthanasia.^102^ Indeed, only a small number of cases of late termination of pregnancy are reported in the Netherlands each year,^103^ and it could be suggested that cases of embryopathic abortion are underreported due to doctors’ unwillingness to invite prosecution for providing medical treatment. Nevertheless, the 2022 LTP Evaluation found no evidence of underreporting. It did, however, note that the potential for prosecution and the degree of uncertainty about the potential for criminal liability often render doctors very reluctant to perform late abortions. It recognized that doctors prefer, instead, to refer pregnant persons to doctors in other jurisdictions, or to perform embryopathically indicated abortions prior to the 24th week as an instance of resolving their emergency situation, thus not triggering a reporting requirement and subsequent scrutiny.^104^ Referrals are generally made to clinics in neighbouring Belgium, where no time limit applies to abortions performed on the basis of an embryopathic indication. Moreover, the criteria for what will constitute a ‘particularly severe and incurable disease’ justifying embryopathic abortion are not defined in the Belgian Act regulating abortion,^105^ providing significantly broader access to embryopathically indicated abortions, including, for example, cases of Down’s syndrome that would not meet the criteria for category 2 in the Netherlands.

A significant problem is uncertainty for treating health professionals, uncertainty that results from the fact that the question of whether the legal requirements have been met is only addressed *after* the occurrence of the termination of pregnancy. There is no mechanism for doctors to apply for certification that their categorization, or actions in terminating a pregnancy are lawful in advance of the termination. Given the enhanced due care criteria applied to category 2 cases (where the fetus is viable),^106^ the determination of whether the case should be categorized as a category 1, or 2 case is crucial. For example, in relation to category 2 cases, the attending doctor is required to obtain a second opinion on the applicability of the due care criteria.^107^ If the assessment committee finds that a doctor wrongly categorized a case as category 1, there is no opportunity to comply with the due care requirement of a second opinion retrospectively. Inevitably, the doctor will fail to comply with a key due care criterium and be referred to the prosecuting authorities.

In the context of abortion, there is nothing similar to the SCEN network, a network of doctors trained in providing independent assessments in relation to termination of life. SCEN has proved very important in the euthanasia context, with the Regional Euthanasia Review Committees consistently recommending the use of a SCEN consultant as a means of ensuring the high quality of the required second opinion.^108^ Professionalization of the consultation process has much to recommend it—SCEN has ensured the availability of highly trained, experienced consultants throughout the country, experts who are able to offer advice, having a thorough knowledge of the statutory requirements relating to euthanasia, and who can also offer support to the attending doctor. Such high-quality consultation is important, and if introduced into the abortion process would function not only as a means of confirming that the requirements for termination of pregnancy are met, but also operate as a form of a priori review, allowing doctors to feel more secure in their decisions.^109^

The retrospective assessment of compliance with the due care criteria creates significant uncertainty for doctors.^110^ Both the 2013 and 2022 evaluations of the late termination of pregnancy regulations recognized that doctors are hesitant to perform late clinically indicated abortions in the Netherlands due to the fear that they could be prosecuted and convicted of a serious criminal offence. The evaluations found that the dual control mechanism applied to category 2 abortions is unduly burdensome and recommended that only cases where the doctor has failed to comply with the due care criteria should be referred to the prosecutorial authorities.^111^ Nevertheless, to date, there has never been a prosecution for a late termination of pregnancy under the Ministerial Regulations.^112^ Seeking to avoid the uncertainty and stigma engendered by a referral to the prosecution authorities, doctors try to make decisions about abortion before the end of the 23rd week of pregnancy, allowing them to take advantage of the exemption from criminal liability set out in Article 296 (5) of the WvS, without having to submit to review, or ‘invite’ prosecution. This highlights the potential impact of revisions to the guideline on perinatal care, because a reduction in the viability threshold could reduce the time available to use the exemption designed to allow assisting a pregnant person in an emergency situation, in an already tight timeframe after the 20-week anomaly scan. For that reason, both the Second Review of the Wafz and 2022 LTP Evaluation recommended that the current 24-week threshold (implicit in the penal code, but explicit in the ministerial regulations relating to late termination of pregnancy) should be retained and *viewed separately* from the treatment threshold applied to neonates.^113^

In cases where it was not possible to terminate the pregnancy within the 24-week limit, the Second Review of the Wafz and 2022 LTP Evaluation noted that doctors sometimes referred patients abroad for terminations, rather than offering abortion in the Netherlands.^114^ This underlines how limited the protection afforded to fetal life by the restrictive and oppressive approach adopted in the Netherlands actually is. It also demonstrates the magnitude of the viability threshold’s potential impact upon pregnant people, disadvantaging those who are unable to travel to obtain an abortion in another jurisdiction, whether due to work and caring commitments, age, or lack of funding, and undermining both the continuity of care and the provision of post-abortion care. The 24-week cut-off point for crisis-based abortions also impacts upon the psychological wellbeing of those affected—pregnant persons may feel compelled to request an abortion before complete clarity is available in relation to the likely severity of the condition and before they have had the time to come to terms with the diagnosis.^115^ Alternatively, they may be left with no alternative to travelling to another jurisdiction in order to access abortion and suffering the associated stigma of undergoing what might in the Netherlands be categorized as an ‘unlawful’ abortion.^116^

##### b. Maternal indication

The ministerial regulation of late terminations of pregnancy^117^ deals solely with abortions based upon an embryopathic indication, post-viability abortions on the basis of a maternal indication fall out with the Wafz, exposing the doctor to criminal liability under both Articles 289 (murder) with 82a, and 296 (abortion) of the WvS concurrently. However, abortion due to a maternal indication *can* be justified by necessity.^118^ According to the Dutch Association for Obstetrics and Gynaecology (NVOG), maternal indications warranting termination of pregnancy to reduce severe maternal morbidity and prevent mortality include hypertensive disorders, severe impairment of cardiac function, rejection of a transplant organ and sepsis.^119^ Generally, in the case of a maternal indication for abortion, labour will be induced. In such cases, the intention is not to kill the fetus and if the child is stillborn no offence will be committed.^120^ If the fetus dies as a result of the termination, the unnatural death must be reported to the municipal pathologist, but no referral to an assessment committee, or the prosecuting authorities is required.^121^

In comparison with embryopathically indicated abortion, abortion based on averting serious harm to the pregnant person’s life or health is subject to relatively few rules and is primarily dealt with by professional guidance.^122^ Clinical expertise is recognized, but in the context of a maternal indication the exercise of that expertise is not subjected to oversight by an independent review commission, or the prosecution authorities. The two indications are distinct: in the case of a maternal indication, there is a claim to self-defence, the pregnant person’s life and/or health is prioritized over that of the fetus.^123^ However, it is clear that this indication is narrowly construed, being limited to physical rather than mental health.^124^ Furthermore, it cannot be used to justify late abortion in circumstances where the pregnant person finds themself in an emergency situation unrelated to an imminent threat to life or health, for example, where they seek a late abortion due to domestic violence. In such cases, the often-vulnerable people will be left with no alternative but to travel to obtain an abortion in another country or to procure abortifacients without medical support.

## III. COMPARATIVE ANALYSIS

Reflecting upon the regulatory frameworks for abortion in England and Wales and the Netherlands, two key themes appear: (i) the use and impact of the criminal law to regulate abortion; and (ii) the framing of abortion as a medical decision aimed at alleviating a crisis, rather than as an individual’s reproductive choice. Central to both themes is the significance attributed to viability by the law. We argue that viability, a concept of uncertain parameters, is ill-suited to, and overemphasized in, the abortion context,^125^ and that abortion has no place in the criminal law. Pregnant people should be empowered to exercise their autonomy and reproductive freedom, rather than be subject to a third-party decision that abortion is permissible in the circumstances, *whatever the stage of pregnancy*.

### A. Criminalization

In both jurisdictions, abortion is constructed as a crime,^126^ albeit with an exception when performed by a doctor in accordance with the AA 1967 (England and Wales) or the Wafz 1981 (the Netherlands). The use of the criminal law to regulate abortion is symbolic, as Andrew Simester and Andreas von Hirsch argue, ‘criminal law has a communicative function which the civil law does not. It speaks with a distinctively moral voice.’^127^ In the context of abortion, this distinctively moral voice over-regulates and exceptionalizes abortion compared to other forms of healthcare.

Abortion (both medical and surgical) is an incredibly common procedure in the UK;^128^ in 2021, there were 214,256 abortions in England and Wales (around 16.8 per 1000 women).^129^ Despite the fact that it is so common, and much more common than other procedures, no other medical procedure is subject to the same level of regulation where detailed requirements are set out concerning where the procedure may be undertaken, by whom and when.^130^ These requirements are not dictated by medical risk, instead, they are designed to underline the idea that abortion is *not* a standard medical procedure.^131^ The fact that abortion is treated differently from other medical procedures ensures that the stigma attached to it endures.^132^ Indeed, the very fact that abortion remains a crime has a chilling effect,^133^ with serious consequences for access. Moreover, as the WHO notes, criminalization can significantly impact upon ‘the provision of quality care’ by suppressing healthcare professionals’ ‘actions due to the fear of reprisals or penalties.’^134^ This is echoed by the 2022 LTP Evaluation’s finding that doctors were reluctant to perform late abortions even when permitted by the regulations, preferring to refer pregnant persons to clinics abroad due to concern about the potential for incurring criminal liability. This concern extends to a misplaced fear of prosecution for referring their patient to a clinic in another jurisdiction where late abortion is lawful.^135^ Such concerns were found not only to limit access to later abortion on embryopathic grounds in the Netherlands, but also to negatively impact upon proper transfer to another doctor and aftercare where pregnant people sought abortion in another jurisdiction.^136^

This chilling effect is particularly pronounced in the Netherlands,^137^ where post-viability abortions fall outside the scope of the Wafz. Article 82a of the WvS extends the scope of the crimes against life delineated in the Penal Code to apply to the viable fetus, making viability the boundary between lawful medical termination of pregnancy and a crime against life. Pre-viability abortion (even in the case of maternal or embryopathic indication) is framed as resolving the pregnant person’s emergency situation and will not require notification to an Assessment Committee, or the prosecution authorities. After viability, their emergency situation will be disregarded in all but the most extreme circumstances, indicating the crucial nature of viability in this context. Post-viability abortion may be permissible in the case of a maternal or a narrowly defined embryopathic indication, but if the fetus is viable such abortions will constitute criminal acts and require justification.

The impact of criminalization was criticized by the Central Expert Committee on Late Termination of Pregnancy Regulations, leading to the recommendation that the Dutch Penal Code should be amended to include an exemption for termination after viability on the basis of an embryopathic (encompassing both categories) and a maternal indication.^138^ Currently, where the doctor complies with the due care criteria, abortion in the case of a threat to the pregnant person’s life or health will be justified by reference to necessity. A criminal investigation will only be initiated if there are grounds to believe that the due care criteria have not been satisfied. Similarly, abortions performed on the basis of fatal fetal anomaly, where crucially the fetus is judged non-viable, will not be referred to the prosecuting authorities if the Assessment Committee is satisfied that the usual due care criteria were fulfilled. However, in the case of non-fatal fetal anomaly, the regulations demand an automatic referral to the prosecution authorities, regardless of the conclusion reached by the committee. Seen in this light, merely conducting an abortion on the basis of fetal anomaly might be perceived as *inviting* review and ultimately prosecution. Unsurprisingly, this has a chilling effect, rendering many doctors reluctant to perform abortions post-viability, to subject themselves to a criminal investigation. The categorization of post-viability abortion as a criminal act is not just empty symbolism. The potential for prosecution *is* real, particularly in the case of category 2 (non-fatal) embryopathic abortion;^139^ the stigma is real.^140^

In England and Wales, viability takes a similarly central role as we outlined earlier because abortion is available on socio-medical grounds before 24 weeks, but after this point it constitutes a crime unless performed to save the pregnant person’s life or avert grave permanent injury to their health, or an embryopathic indication. It is important to note that the embryopathic indication is comparatively broad in England and Wales, especially when compared to the Netherlands; it requires a substantial risk that the fetus will be ‘handicapped’, rather than ‘no reasonable doubt’ about the prognosis and diagnosis.^141^ Despite requiring only that the doctors form the opinion in good faith that there is a substantial risk the child will be ‘seriously handicapped’, cases such as *Jepson* and *Crowter* have underlined the ill-defined nature of the indication. In 2022, the Court of Appeal reasoned that doctors are used to making decisions assessing risk and degree of disability and that ‘Parliament's use of broad concepts such as “substantial risk” and “serious handicap” properly reflects that context’.^142^ However, the British Medical Association has argued that doctors are anxious ‘about the risk of criminal prosecution if their clinical judgment is challenged in relation to a [post-viability] abortion’, leading to concern that pregnant persons are ‘sometimes encouraged to make decisions before the 24-week time limit’.^143^

The viability threshold is particularly burdensome in the non-lethal fetal anomaly context in both jurisdictions. It is not until 20–22 weeks’ gestation that the fetal organs are sufficiently developed to enable anomalies to be identified by ultrasound. If terminations are to be performed before 24 weeks, little time is available to the pregnant person to seek advice and counselling if desired, to understand the nature of the fetal anomaly, and make a decision about termination. Moreover, in some cases clarity about the diagnosis and prognosis may not be achieved before the 24th week, leading to pressure to make a decision without the full facts before the artificial deadline that results from professionals’ concerns about the potential for criminal liability in the case of late termination of pregnancy.^144^ The chilling effect of the criminal law is evident in both jurisdictions; pregnant people are pressured into making quick decisions in order to ensure they have a pre- rather than post-viability abortion. Where it is too late for this, some people find themselves having to travel abroad to access abortion or may source medication online to end their pregnancies without medical assistance; others will find themselves forced to continue the pregnancy to term.^145^

While viability is determinative of the relative ease of access to abortion (or indeed, if it is permissible at all), this threshold only applies to cases of medical termination of pregnancy. Where the abortion is induced *outside* the scope of the Act, it is criminalized from implantation onwards. Significantly, and unlike the Dutch law, the pregnant person themselves may commit the offence of procuring their own miscarriage in England and Wales (section 58 OAPA 1861), or later in pregnancy the offence of child destruction (section 1 of the ILPA 1929), an important point given the wide-scale accessibility of abortion pills on the internet.^146^ Recently, there has been a significant increase in individuals being charged with procuring a miscarriage for sourcing and administering abortifacients without medical support.^147^ In such cases, viability is regarded as an aggravating factor and the pregnant person is framed as an aggressor. This characterization is evident in the sentencing of Sarah Catt, who bought misoprostol online after failing to obtain an abortion at two clinics due to the late stage of her pregnancy. Upon sentencing her to eight years imprisonment, Cooke J stressed:

The gravamen of this offence is that, at whatever stage life can be said to begin, the child in the womb here was so near to birth that in my judgement all right thinking people would consider this offence more serious than manslaughter or any offence on the calendar other than murder.^148^

Representing the ‘strong arm of the law’, cogent reasons are required to justify the use of criminal law to regulate any area of law and its use is particularly disproportionate in the case of a procedure framed, in both jurisdictions considered here, as medical treatment.^149^ The protection afforded to the fetus by the criminal law is largely symbolic; while subject to monitoring, abortion remains widely available up to viability in both jurisdictions. However, the symbolism is important, as Rebecca Cook has argued, ‘By framing abortion as a crime societies ascribe deviance to those seeking and providing it.’^150^

Similarly, the protection afforded to pregnant persons’ life and health through the criminalization of abortion is limited. This is exemplified in those circumstances where impacted pregnant persons are left with no option but to source a termination outside the jurisdiction. Where abortions are sought in other jurisdictions, pregnant persons often have more limited opportunities for follow-up care and may find it distressing to complete the termination abroad and then potentially bring home the remains of their fetus, or commence the termination abroad, travelling home while it is in progress. Alternatively, abortion-seekers must avail themselves of unlawful opportunities to purchase abortion pills online. The continued regulation of abortion as a crime acts as a barrier to access, engendering uncertainty and fear on the part of doctors at the boundary of viability and stigmatizing those seeking to exercise their reproductive liberty.^151^ In its 2022 abortion care guidelines, the WHO noted the problem of stigma arising from criminalization and recommended the full decriminalization of abortion by:

removing abortion from all penal/criminal laws, not applying other criminal offences (e.g., murder, manslaughter) to abortion and ensuring there are no criminal penalties for having, assisting with, providing information about, or providing abortion, for all relevant actors.^152^

On the international stage, there have been increasing calls to decriminalize abortion due to the harm that arises from its regulation as a crime.^153^ Thus, the United Nations Special Rapporteurs have repeatedly called for states to decriminalize abortion.^154^ Both the Netherlands and the UK have ratified the UN Convention on the Elimination of All Forms of Discrimination Against Women (CDEAW), that specifies that women should have the right to control their reproduction: ‘the same rights to decide freely and responsibly on the number and spacing of their children and to have access to the information, education and means to enable them to exercise these rights.’^155^ In condemning the United Kingdom for a legal framework that only allowed abortion in Northern Ireland where necessary to save the pregnant person’s life, the Committee on CDEAW found that ‘criminalization of abortion amounts to discrimination against women’.^156^ The legal framework in question was sections 58–59 of the OAPA 1861,^157^ which has now been repealed in Northern Ireland but is still the basis of the law on abortion in England and Wales. We suggest that the time has come for both jurisdictions, and others across the globe, to follow the lead of jurisdictions in Latin America (Argentina, Colombia, Mexico),^158^ to remove abortion from the ambit of the criminal law^159^ and to subject it to the normal rules applicable to medical treatment. As Herring and others have explained: ‘abortion services are already (and would remain) subject to a dense web of other regulation, including general provisions of criminal and civil law, licensing and inspection requirements, and professional oversight.’^160^ Thus, decriminalization abortion would still be appropriately regulated, rather than over-regulated.

### B. The medicalization of abortion and disregard for autonomy

Both jurisdictions characterize abortion as a medical solution to a ‘crisis’ pregnancy up to viability, foregrounding medical authority rather than human rights and devolving abortion decisions to the medical profession. In framing abortion entirely as a medical matter, rather than a broader matter of controlling one’s own reproductive freedom (whether conceptualized as the right to bodily integrity, or liberty to decide whether and how to reproduce), access to abortion remains fragile. In adopting a medical model of regulation, both jurisdictions fail to recognize a role for pregnant people’s autonomy beyond their ability to request an abortion and the necessity that they consent to the procedure. This stands in direct contention with WHO’s recommendations that abortion be available on request, and that legal frameworks should not ‘restrict abortion by grounds,’^161^ or gestational time limits.^162^

Abortion is *exceptionalized—*subjected to additional rules outside the general legal framework applicable to medical treatment—in both legal frameworks.^163^ There are no good clinical reasons to separate out abortion as distinct from other areas of healthcare; the procedure is very safe and there are no legitimate justifications for treating abortion medications and procedures as distinct from other healthcare interventions with similarly low levels of risk/invasiveness. While some may argue that there are justifications for treating abortion ’differently,’ such conceptions of the treatment are about moral perceptions of the appropriateness of the treatment.^164^ The exceptionalism in treating abortion differently is stigmatizing to both the pregnant person (who is framed as requiring assistance to make a careful choice and whose choice is subject to validation by a third party (the doctor applying a set of legal rules)) and the doctor who terminates the pregnancy.^165^

Abortion is widely available prior to viability and funded by the state in both jurisdictions, however the procedural requirements in both Dutch and English law emphasize that it is not simply a reproductive choice, but a medical solution, one that uniquely requires state supervision.^166^ These procedural requirements, doing both the work of ‘moralising’ the choice to have an abortion and the work of helping people have abortions, have significant impacts on the quality of care in a multitude of ways,^167^ including, for example, delaying access for bureaucratic reasons. That pre-viability abortions are widely available in both jurisdictions is the result of the liberal approach taken by doctors to abortion, minimizing the impact of the substantive and procedural requirements on individuals needing abortion.^168^ However, despite the availability of abortion, reproductive freedom is fragile because it is dependent upon medical benevolence rather than the right to control one’s own body and reproductive future.

The wording of the Dutch Act emphasizes that it is not the individual’s desire to terminate their pregnancy but their emergency situation that renders abortion permissible. Similarly, Sally Sheldon’s work analysing the passage of the AA 1967 identified that only ‘3 images of femininity … [were] presented in the debates: the [pregnant person] as minor, as victim and as mother.’^169^ In each case, it was argued that the pregnant person was unable, or unfit, to elect an abortion alone. The procedural requirements imposed in the Acts in both jurisdictions emphasize the pregnant person’s lack of agency. The requirement that two doctors agree that an abortion is permissible in England and Wales,^170^ or that a doctor determines that there is no alternative means of resolving the individual’s emergency situation than to terminate the pregnancy in the Netherlands, serve only to delay access to abortion and to underline that pregnant people are judged incapable of making the decision to have an abortion for themselves while professional judgement is preserved and prioritized.^171^

The very limited role ascribed to autonomy is disregarded once the fetus achieves viability, even though people can experience pregnancy during the third trimester as a crisis situation in exactly the same way as prior to viability. It is the *pregnancy* and not the gestational age of the fetus that constitutes the emergency. The British Pregnancy Advisory Service conducted research into the reasons for ‘late’ abortions and found that rarely was this due to delayed decision-making.^172^ Generally, respondents had only recently become aware that they were pregnant (because they were using contraception, breastfeeding, or had irregular periods), and some were vulnerable because of domestic violence, or were teenagers.^173^ We argue that the viability threshold that prevents such people from accessing safe and legal abortion is inappropriate and stigmatizing. It is also based upon a false premise as there is no evidence that removing the viability threshold would lead to an increase in later term abortions in either jurisdiction,^174^ but it would, we suggest, lead to better and safer care for those seeking abortion by obviating the need to travel abroad or to obtain abortion pills online and risking prosecution.

### C. Viability

The saying ‘timing is everything’ proves particularly true in the case of abortion regulation where time defines the boundary between criminal abortion and permissible medical practice. Both jurisdictions start from the position that the fetus is not a legal person,^175^ but have taken an incrementalist approach to regulating abortion. In the Netherlands, this has been taken to an extreme as Article 82a of the WvS extends the scope of the crimes against life to apply to a fetus. It thus conflates the fetus with a newborn^176^ and eschews the qualifying characteristic applied to homicide offences in England and Wales—that the person killed must have been born alive. By comparison, although the ILPA creates the offence of child destruction in England and Wales, it does not treat the fetus as synonymous with a child. Indeed, the offence of child destruction is a specific offence created precisely to protect ‘the child capable of being born alive,’ rather than extending the ambit of the offences of murder or infanticide. Moreover, even though the maximum penalty for child destruction is life imprisonment, unlike the sentence for murder, it is not a mandatory life sentence; thus, clearly distinguishing between the ‘killing’ of a fetus and a person extant.

In both jurisdictions, viability represents the cut-off point beyond which a pregnancy cannot be terminated unless it poses a threat to the pregnant person’s life or health or is embryopathically indicated. It might be argued that viability is a ‘compromise position’ because it allows abortion on broader grounds before viability and then only on strict therapeutic grounds afterwards. However, such a conceptualization fails to recognize the incongruity of applying an unrelated medical concept (one that is used to estimate the likelihood of survival if the fetus were to be born at a given point in time) to determining whether an individual can terminate an unwanted pregnancy. Furthermore, placing significant emphasis on the different ‘justifications’ for abortion earlier and later in a pregnancy does a disservice to reproductive autonomy: specifically, in failing to recognize that there is a need for abortion later in pregnancy for ‘a broad range of reasons.’^177^ It disregards the lived experiences of persons experiencing difficult pregnancies or pregnancy in difficult circumstances. The WHO explicitly recommends against laws/other regulations prohibiting abortions based on viability (or gestational age limits)^178^ and yet both England and Wales and the Netherlands continue to do so.

#### i. The construct of viability: definition and parameters

##### a. Legal certainty

The need for certainty is particularly important in criminal law where the liberty of the individual is at stake. However, reliance upon viability in the regulation of abortion breaches this requirement both by its inherent vagueness and the uncertainty it creates in the application of the law. We argue that references to viability, whether as a general concept as in Article 82a of the WvS and the ILPA (albeit with a rebuttable presumption of viability set at 28 weeks), or as a particular point in time (24 weeks) in the AA, fail to recognize the inherently uncertain boundaries of viability. Article 82a of the WvS describes viability as being present when the ‘fetus can reasonably be expected to have the ability to survive outside the mother’s body.’ Similarly, section 1 of the ILPA refers to ‘a child capable of being born alive.’ Both of these phrases require clarification—*how long must an entity survive after birth*? The Dutch Supreme Court has stated that it is not necessary to expect the neonate to survive for a lengthy period of time to fulfil the criteria of Article 82a of the WvS,^179^ while the English Court of Appeal noted that an integral characteristic of a ‘child capable of being born alive’ is the ability to breathe (with or without the support of a ventilator).^180^ It would appear that both Acts require little more than the *capacity* for live birth, with no requirement that the child once born alive (taking at least one breath) have the capacity for longer-term survival. However, the meaning attributed to both provisions is subjective and has the potential to change with technology.^181^

Neonatal viability is a medical assessment and depends upon many variables, including the size, weight sex and gestational age of the fetus, the geographic location of the pregnant person, and the medical facilities available in that location.^182^ Clinicians assess whether a particular fetus is likely to survive outside the uterus. Thus, viability is fetus specific, rather than a general measurement capable of application to all pregnancies. It is also an assessment that changes over time, with developments in perinatology enhancing the ability to support survival at ever earlier gestations.^183^ Viability, therefore, represents not a determinate line, but a flexible boundary, rendering it ill-suited to the function of a legal bright line that legislatures seem to imagine.

##### b. The disconnect between viability as a threshold for neonatal treatment and a threshold for lawful abortion

The current boundaries for socio-medical abortion and active treatment for extremely premature neonates roughly converge, but abortion and perinatal care are very different forms of medical treatment. In abortion, the fetus is not intended to survive, whereas in the case of (extremely) premature neonates, all medical care is focused upon trying to ensure its survival. In the case of an unwanted pregnancy, the expectation will generally be that if the pregnancy continues, the child will be born healthy at full term. In the case of an extremely premature neonate, the child is born with all the health problems prematurity entails and a decision will have to be taken about whether active care should be provided.^184^ The danger is that as the threshold for active treatment reduces in line with technological developments, it may be argued that the threshold for ‘social’ abortions should be reduced in line with viability. We argue that the two thresholds are distinct and that the status of the fetus should not be conflated with that of a neonate.

From a clinical perspective, the concept of viability is uncertain. Developments in perinatology have enhanced the ability to support survival at even earlier gestations, but it is important to recognize that even those born alive will not necessarily remain alive for long; many will be too physiologically immature to survive longer-term, and many may experience (common) fatal complications in neonatal intensive care. Moreover, considering viability as a concept denoting whether a child is likely to be able to survive birth is an oversimplification. Viability is not a binary issue, as Leo Han and others have argued, ‘A meaningful discussion of neonatal viability includes not just survival, but honest and candid dialogue with the pregnant person and [their] family about what kind of life to expect.’^185^ Both jurisdictions accept that abortion can be justified in cases of non-fatal fetal anomaly and so recognize that the capacity for survival alone will not suffice as a justification for prohibiting abortion. However, there seems to be a mismatch in the protection afforded to the fetus, in comparison to the newborn, particularly in the Netherlands. The second Dutch category is much more narrowly drawn than its English comparator, as it requires a condition that will lead to a serious and irreparable functional disorder. The fact that cases exist where abortion cannot lawfully be provided, but where doctors might determine that an extremely premature neonate should not be treated, underlines the fact that termination and active treatment of neonates are distinct. The fetus in utero should not be given greater protection than its neonatal counterpart.

The embedding of fetal viability in the law on abortion is ethically problematic. Viability is a medical concept developed to determine when active treatment of a premature neonate is appropriate, not to determine whether a pregnancy can be terminated. The two situations are entirely distinct,^186^ a fact reflected in the legal status accorded to the entity in question. While the fetus at 24 weeks’ gestation *may* survive birth, the fact that it is physically located inside a person with their own rights and interests is highly significant. Unlike the neonate, the fetus at 24 weeks’ gestation has not made morally significant biological adaptations to the environment,^187^ it has no independent existence and is functionally integrated into the pregnant person.^188^ Nevertheless, a viability threshold does not seek to balance those interests; instead, it emphasizes the potential for life of the fetus, elevating the biological status of the fetus to trump the pregnant person’s autonomy, bodily integrity and dignity interests in all but the most extreme circumstances. However, the mere imposition of a viability threshold does little to protect fetal life in practice, rendering its value primarily symbolic. It creates access barriers and bottlenecks as pregnant people and their doctors try to terminate a pregnancy prior to the threshold or refer pregnant people to other countries where late abortions are available. Desperate people, such as Sarah Catt, unable to access abortion via the medical process, purchase abortion pills online and risk criminal sanction.^189^ The use of the viability threshold makes it more difficult to access late abortion, disproportionally impacting upon the vulnerable, the young and those lacking the financial means or the knowledge of how to access abortion after viability. In short, the viability threshold does little to protect fetal life, but endangers pregnant people’s health.

## IV. CONCLUSION

As Leah Eades has noted, the 24-week fetus can be located in multiple settings ‘within a womb, an incubator, or an abortion clinic.’^190^ We argue that the distinctiveness of those settings is crucial, and that neonatal viability has no role to play in the abortion context. Inevitably, framing abortion as a medical matter allows medical concepts to ‘creep’ into regulation. Scientific and medical developments inform the law, but science takes no account of the relational context of pregnancy. Rather, it focuses upon the potential for survival of the fetus, while disregarding the impact continuance of the pregnancy will have upon the pregnant person’s life. By placing viability at the centre of abortion regulation, pregnant people’s rights and interests are disregarded; their emergency situation/personal circumstances are trumped by the biological status of the fetus.

By continuing to anchor the regulation of abortion in the criminal law, both jurisdictions perpetuate the stigmatization of abortion, causing doctors to act cautiously around the threshold of viability for fear of prosecution. They stigmatize those who present later for abortion, leaving them to seek assistance abroad or online. The opportunity costs associated with regulating abortion in the context of criminal law are significant. Criminalized abortion restricts access to care and results in stigma, distress, anxiety, poor quality post-abortion care and a lack in continuity of care. In addition, criminalized abortion imposes financial costs and burdens upon those required to travel to access abortion and increases the workload of healthcare professionals required to submit their clinical decisions to external scrutiny to demonstrate compliance with the regulation.^191^ The increasing ability to bypass controls intended to restrict abortion renders such control illusionary. Criminal regulation of abortion structured around fetal viability does little to protect fetal life but renders abortion less safe, posing a risk to pregnant people’s lives and health.

As developments in perinatology continue to enhance the ability to support survival at ever earlier gestations, it is conceivable that one day even very early abortions would be banned if viability continues to operate as a threshold. We suggest that the time has come to recognize the impact of neonatal viability upon the unrelated medical termination of pregnancy, to decentre viability and to focus instead upon empowering people to make reproductive choices that reflect their own interests.

## Footnotes

1 On the importance of time in medical law see J Harrington, ‘Time as Dimension of Medical Law’ (2012) 20 Medical Law Review 491, and more specifically in the context of abortion and human rights, see J Erdman, ‘Theorizing Time in Abortion Law and Human Rights’ (2017) 19 Health Human Rights 29.

2 J Glover, *Causing Death and Saving Lives* (Penguin 1990) 124.

3 A Zaitchik, ‘Viability and the Morality of Abortion’ (1981) 10 Philosophy & Public Affairs 18; M Hawking, ‘The Viable Violinist’ (2016) 30 Bioethics 312; D Jensen, ‘Birth, Meaningful Viability and Abortion’ (2015) 41 JME 460.

4 Some exceptions: EC Romanis, ‘Is “Viability” Viable? Abortion, Conceptual Confusion and the Law in England and Wales and the United States’ (2020) 7 Journal of Law Biosciences <doi.org/10.1093/jlb/lsaa059>; L De Proost and others, ‘The Edge of Perinatal Viability: Understanding the Dutch Position’ (2021) 9 Frontiers in Pediatrics 634290; Erdman (n 1).

5 See Romanis (n 4) 3–4.

6 De Proost (n 4) 3.

7 P Singer, *Practical Ethics*, 3rd edn (CUP 2011) 126.

8 IG Cohen and S Sayeed, ‘Fetal Pain, Abortion, Viability and the Constitution’ (2011) 39 Journal of Law, Medicine & Ethics 235, 237.

9 World Health Organization, ‘Abortion Care Guideline’ <https://www.who.int/publications/i/item/9789240039483> 28, accessed 16 March 2023.

10
*Tweede evaluatie Wet afbreking zwangerschap*, 2020 (Second Review of the Termination of Pregnancy Act), hereinafter Second Review of the Wafz, 11, 62ff; D Wilkinson, ‘Lifesaving Treatment for Babies Born at 22 Weeks doesn’t Mean Abortion Law should Change’, *The Conversation* (2019) < https://theconversation.com/lifesaving-treatment-for-babies-born-at-22-weeks-doesnt-mean-abortion-law-should-change-125845> accessed 16 March 2023.

11 De Proost (n 4).

12 Second Review of the Wafz (n 10) 11, 62ff.

13 S de Zordo and others ‘Gestational Age Limits for Abortion and Cross-border Reproductive Care in Europe: A Mixed-methods Study’ (2020) 128(5) BJOG 838.

14 WHO (n 9) 28. Specifically referring to the context in England and Wales see S Sheldon, ‘The Decriminalisation of Abortion: An Argument for Modernisation’ (2016) 36 Oxford Journal of Legal Studies 345, 334–65; BPAS, ‘*But I was using contraception ….’ Why Women Present for Abortions After 20 Weeks* (2017) <https://www.bpas.org/media/2027/late-abortion-report-v02.pdf> accessed 16 March 2023, and in the Netherlands see Second Review of the Wafz (n 10).

15 WHO (n 9).

16 Eg, Special Rapporteur on the right of everyone to the enjoyment of the highest attainable standard of physical and mental health, Interim Report to the General Assembly (2011) (UN Doc A/66/254); Special Rapporteur on torture and other cruel, inhuman or degrading treatment or punishment, Report to the Human Rights Council (2016) (UN Doc A/HRC/31/57).

17 Note that a doctor is also guilty even if the person is not pregnant. The pregnant person can also commit this offence by procuring their own miscarriage if they were pregnant. See, for example, *R v Sarah Louise Catt* (2012), unreported; *R v Natalie Towers* (2015), unreported.

18 From implantation *(R (Smeaton) v Secretary of State for Health* [2002] EWHC 610 (Admin)) to term.

19 ILPA 1929, s 1(2).

20 A Grubb, ‘Abortion Law in England: The Medicalization of a Crime’ (1990) 18 Law Medicine & Healthcare 146, 149; Romanis (n 4) 5.

21 AA 1967, s 1 (1) (a)–(d).

22 ibid, s 1 (2).

23 E Cave and M Brazier, *Medicine, Patients and the Law* (Manchester UP 2016) 404.

24 ibid; E Jackson, *Regulating Reproduction: Law, Technology and Autonomy* (Hart 2001) 80.

25 BMA, *The Law and Ethics of Abortion: BMA views*, 2020 [2.1.5]; Royal College of Obstetricians and Gynaecologists, *Submission to the House of Commons Science and Technology Committee Abortion Inquiry* (2006) [2.1.1]; E Raymond and D Grimes, ‘The Comparative Safety of Legal Induced Abortion and Childbirth in the United States’ (2012) 119 Obstetrics & Gynecology 215.

26 P Lohr and others, ‘How would Decriminalisation Affect Women’s Health?’ in S Sheldon and K Wellings (eds), *Decriminalising Abortion in the UK* (Policy Press 2020).

27 JA Parsons and EC Romanis, *Early Medical Abortion, Equality of Access, and the Telemedical Imperative* (OUP 2021) 17.

28 ibid.

29 Romanis (n 4) 5.

30 A Grubb, ‘The New Law of Abortion: Clarification or Ambiguity?’ (1991) Criminal Law Review 659, 661.

31 Romanis (n 4) 11.

32 ibid 5.

33 Grubb (n 20) 149; J Keown, ‘The Scope of the Offence of Child Destruction’ (1988) 104 *LQR* 120, 123ff. Homicide has always, in English law, been restricted to those born alive, E Coke *Institutes* III, 1648, 50, and affirmed by the House of Lords in *Attorney-General’s Reference (No 3 of 1994)* [1998] AC 245.

34 ILPA 1929, s 1(1).

35 Romanis (n 4) 7. See also J K Mason, *The Troubled Pregnancy: Legal Rights and Wrongs in Reproduction* (CUP 2007) 21; EC Romanis, ‘Challenging the ‘Born Alive’ Threshold: Fetal Surgery, Artificial Wombs, and the English Approach to Legal Personhood’ (2020) 28 Medical Law Review 93–123.

36 Grubb (n 30) 663.

37 See, for example, *R v Davison* (2021) unreported; *R v Wilson* [2017] 1 Cr App R (S). But note *R v Mohammed* (2007) unreported, where an individual was convicted of child destruction following a termination at 34 weeks gestation. It was not established who had terminated the pregnancy.

38
*C v S* [1988] 1 QB 135; *Rance and Another v Mid-Downs Health Authority and Another* [1991] 1 QB 587.

39
*R v Bako* (2015), unreported.

40 See, for example, *R v Davison* (2021) unreported: 29 weeks; *R v Wilson* [2017] 1 Cr App R (S): 32 weeks; *R* v *Mohammed* (2007) unreported: 34 weeks.

41 Grubb (n 20) 150; Romanis (n 35) 116.

42 AA 1967, s 1(1)(b).

43 The examples given in the HL debates: HL Vol 522 Col 1039.

44 s 1(1)(d) AA 1967.

45 RCOG, *Termination of pregnancy for fetal abnormality in England, Scotland and Wales*, 2010, 9; BMA (n 25) [2.1.4].

46 See for example S McGuinness, ‘Law, Reproduction, and Disability: Fatally “Handicapped”?’ (2013) 21 Medical Law Review 213; R Scott, ‘Interpreting the Disability Ground of the Abortion Act’ (2005) 64 Cambridge Law Journal 388, 396.

47
*Crowter v Secretary of State for Health and Social Care* [2021] EWHC 2536. The claimants sought a declaration of incompatibility in respect of s 1(1)(d) AA 1967, arguing that it is incompatible with arts 2, 3, 8 and 14 of the European Convention on Human Rights because it differentiates between pregnancies where there is a substantial risk that, if born, a child would be ‘seriously handicapped’ and pregnancies where it would not, permitting only the former category to be terminated after viability absent a medical indication. Although the claimants focused upon Down’s syndrome, they argued that this distinction would be impermissible in any case where a risk of ‘serious handicap’ was used. The court dismissed the claim, finding that the focus of s 1(1)(d) is the rights of the pregnant person and their medical treatment, rather than the fetus even after viability [102]. For an excellent analysis of this decision see Z Tongue, ‘*Crowter v Secretary of State for Health and Social Care* [2021] EWHC 2536: Discrimination, Disability, and Access to Abortion’ (2021) 30 Medical Law Rev 177. Moreover, as the Supreme Court recognized in *Re Northern Ireland Human Rights Commission’s Application for Judicial Review* [2018] UKSC 27, ‘the current legislation already recognises important limitations on the interests and protection of the unborn fetus. It permits abortion of a healthy fetus in circumstances where the mother’s life would be at risk or where she would suffer serious long-term damage to her physical or psychological health. There is therefore no question of any absolute protection of even a healthy fetus,’ [119] *per* Lord Mance.

48
*Jepson v Chief Constable of West Mercia Police* [2003] EWHC 3318 (Admin). Jepson sought judicial review of the decision not to prosecute doctors who had terminated a pregnancy involving a viable fetus with a cleft palate. Following a police investigation, the West Mercia Chief Crown Prosecutor confirmed that on the evidence the doctors had formed the necessary opinion in good faith and that there should be no charges against them.

49 RCOG (n 45) 8.

50 ibid.

51 ibid 9.

52 BMA (n 25) [2.1.1]; This is discussed further below, Section IV.

53
*Wetboek van Strafrecht.*

54
*Wet afbreking zwangerschap.*

55
*Besluit afbreking zwangerschap.*

56 Kamerstukken II 1978/79, 15475, 3 *Regelen met betrekking tot het afbreken van zwangerschap (Wet afbreking zwangerschap)* (Explanatory Memorandum, Regulations regarding the Termination of Pregnancy (Wafz)), 9–10.

57 Explanatory Memorandum (n 56) 9–10, 15–17. The Explanatory Memorandum to the Act explains that: ‘Induced abortion is a measure that can only be justified by the needs of [pregnant persons]. However, we do not consider it possible to define these emergencies in the law. The circumstances that can justify the termination of a pregnancy in individual cases cannot be translated into general terms, let alone recorded in a legal formula’, 15–16.

58 The premise of the Act is that abortion will result from an unwanted pregnancy; that only unwanted pregnancies are terminated. However, where a pregnancy is terminated due to a risk to the pregnant person’s health or life, or because of fetal anomalies, the pregnancy was usually desired, or at least the pregnant person had decided to continue the pregnancy.

59 art 296 (1) WvS.

60 ibid

61 art 296 (5) WvS.

62 art 5(2)(b) Wafz.

63 art 5(2)(c) Wafz.

64 art 5(2)(a) Wafz.

65 art 3 Wafz; the Act designated a reflection period of five days except where necessary to save life, but this was subject to sustained criticism, see for example *Evaluatie Wet afbreking zwangerschap* (Review of the Termination of Pregnancy Act) 2005, 158; Second Review of the Termination of Pregnancy Act 2020 (n 10) 83–86; J de Bree, ‘*Bezint eer ge begint: de beraadtermijn voor abortus in gezondheidsrechtelijk perspectief*’ (2021) 45(4) *Tijdschrift voor Gezondheidsrecht* 374. In 2022, an amendment was passed, replacing the fixed reflection period with a flexible period, allowing doctors and pregnant persons to determine the appropriate length of time in the circumstances, VAO Pregnancy and Birth, AO 10/12.

66 Explanatory Memorandum (n 56) at [23].

67 arts 289, 82a WvS.

68 art 296 WvS.

69 If the pregnant person induces the abortion themselves, they are liable to prosecution for child murder, or infanticide, arts 290, 291, 82a WvS. The pregnant person is excluded from the ambit of art 296, cf s 58 OAPA 1861 where the pregnant person is one of the addressees of the prohibition.

70 Explanatory Memorandum (n 56) 32–33. Similarly, art 2 Burial and Cremation Act 1991 (*Wet op de lijkbezorging*) defines stillbirth as a fetus born at a gestational age of at least 24 weeks, or at an earlier gestation if it survives more than 24 hours after birth, recognising that viability may occur below 24 weeks.

71 NJ 1991, 217: HR, May 29, 1990, No 87203.

72 ibid 5.1.

73 Second Review of the Wafz (n 10) 60.

74 F Söderström and others, ‘Outcomes of a Uniformly Active Approach to Infants Born at 22-24 Weeks of Gestation’ (2021) 106 Archives of Disease in Childhood. Fetal and Neonatal Edition 413.

75 De Proost (n 4).

76 MW de Laat and others, ‘Richtlijn “Perinataal beleid bij extreme vroeggeboorte”’ (Guideline ‘Perinatal care for extremely premature neonates’) (2010) Ned Tijdschr Geneeskd 1.

77 Second Review of the Wafz (n 10) 67; *Evaluatie Regeling beoordelingscommissie late zwangerschapsafbreking en levensbeëindiging bij pasgeborenen* (Evaluation of the Assessment Committee Regulations for Late Termination of Pregnancy and Termination of Life in Newborns), 2022 (hereinafter LTP Evaluation 2022) recommendation 1, 129.

78 art 5 Wafz.

79 ibid.

80 The Act also sets out what will constitute good aftercare, requiring for example that after the abortion the pregnant person should be provided with contraceptive advice as well as a follow-up examination and any medical treatment she requires, art 5 Wafz.

81 S Halliday, ‘Protecting Human Dignity: Reframing the Abortion Debate to Respect the Dignity of Choice and Life’ (2016) 13(4) Contemporary Issues in Law 287; Parsons and Romanis (n 27).

82 Cf s 1(1)(c) and (b) Abortion Act 1967, respectively.

83 Cf s 1(1)(d) Abortion Act 1967.

84 Post-viability abortions will constitute a crime both under Article 296 WvS (abortion) and other crimes against life (arts 289, 290, 291 WvS – homicide, child murder and infanticide).

85 art 40 WvS.

86 S Gevers, ‘Third Trimester Abortion for Fetal Abnormality’ (1999) 13 Bioethics 306, 312.

87 Minister of Security and Justice and the Minister of Health, Welfare and Sport *Regeling beoordelingscommissie late zwangerschapsafbreking en levensbeëindiging bij pasgeborenen*, 11 December 2015, reference 885614-145412-PG.

88 ibid art 1.

89 Second Review of the Wafz (n 10) 46, 60; ZonMW *Evaluatie van de Regeling centrale deskundigencommissie late zwangerschapsafbreking en levensbeëindiging bij pasgeborenen* (Evaluation of the Central Expert Committee on Late Termination of Pregnancy Regulations in a category 2 case and termination of life in neonates), 2013, 12, 68–69, 74; *Evaluatie Regeling beoordelingscommissie late zwangerschapsafbreking en levensbeëindiging bij pasgeborenen* (Evaluation of the Assessment Committee Regulations for Late Termination of Pregnancy and Termination of Life In Newborns), 2022, see especially chapter 3.

90 art 5 Regulations (n 87).

91 The justification of necessity (art 40 WvS) will apply if the doctor has complied with the specific category 2 due care criteria.

92 art 6 Regulations (n 87).

93 ibid.

94 LTP Evaluation 2022 (n 77) 3.51.

95 ibid 4.42.

96 ibid 4.42, table 4.1.

97 ibid 3.4.1, table 3.7.

98 In contrast, the review committees that assess compliance with the due care criteria for euthanasia are only required to refer cases of non-compliance, art 9(2) Termination of Life on Request and Assisted Suicide (Review Procedures) Act 2001.

99 If the Committee finds that the doctor has acted in accordance with the due care criteria, there will, in principle, be no reason for the prosecuting authorities to investigate or prosecute the doctor, *Aanwijzing vervolgingsbeslissing inzake late zwangerschapsafbreking en levensbeëindiging bij pasgeborenen* (2017A003) (Prosecution decision regarding late termination of pregnancy and termination of life in newborns), 2017, 4.2. Significantly, just as in cases regarding euthanasia, the BPG have made it clear that even where the procedural elements of the due care criteria have not been complied with, the justification of necessity can still apply. Therefore, the principal consideration will be whether a substantive requirement has been met, for example, the requirement that the doctor was convinced that the fetus was suffering hopelessly, Prosecution Decision.

100 LTP Evaluation 2013 (n 89) 65–66; LTP Evaluation 2022 (n 77), see especially 9–14, 80, 115.

101 The 1998 reporting procedure for euthanasia cases required that all cases of euthanasia and assisted suicide be referred to the prosecution authorities. See further S Halliday, ‘Regulating Active Voluntary Euthanasia: What can England and Wales Learn from Belgium and the Netherlands’ in A Garwood-Gowers and others (eds), *Contemporary Issues in Healthcare Law and Ethics* (Elsevier 2005) 281ff; S Halliday, ‘Comparative Reflections upon the Assisted Dying Bill 2013: A Plea for a more European Approach’ (2013) 13 Medical Law International 135.

102 See further Halliday 2005 (n 101) and 2013 (n 101).

103 LTP Evaluations 2013 and 2022 (n 89 and n 77). In 2018, 11 reports of late termination of pregnancy categories 1 and 2 were received by the Committee, LZA/LP beoordelingscommissie late zwangerschapsafbreking en levensbeëindiging bij pasgeborenen *Jaarverslag 2021* (Assessment Committee for Late Termination of Pregnancy and Termination of Life in Newborns Annual Report 2021), 2022, [2.2]. In contrast, in 2019, 2020 and 2021 only three, four and seven reports respectively were received, statistics more consistent with the number of reports in 2016 and 2017, LZA/LP *beoordelingscommissie late zwangerschapsafbreking en levensbeëindiging bij pasgeborenen Jaarverslag 2021* (Assessment Committee for Late Termination of Pregnancy and Termination of Life in Newborns Annual Report 2021), 2022, [2.1].

104 LTP Evaluation 2022 (n 77) 9–14, 5.54, 5.5.

105 art 2, 5°Belgian Act on the Voluntary Termination of Pregnancy. For a detailed analysis of the Belgian law, see F de Meyer, ‘Late Termination of Pregnancy in Belgium: Exploring its Legality and Scope’ (2020) 27 European Journal of Health Law 9.

106 art 6 Regulations (n 87).

107 Noticeably, there is no requirement that the second opinion confirms the view of the attending doctor. However, if a doctor were to perform a late termination of pregnancy following a non-confirmatory second opinion, that would undoubtedly operate as a red flag to the Assessment Committee and the 2022 LTP Evaluation found that in such circumstances doctors were unlikely to offer a termination (n 77) 71.

108 The preference for a second opinion provided by a SCEN physician is set out in the Regional Review Committee’s *Euthanasia Code 2022: Review Procedures in Practice*, 2022, 27. See further Halliday 2005 (n 101) and 2013 (n 101).

109 See further Halliday 2013 (n 101).

110 A further source of insecurity can be attributed to the different way in which late termination of pregnancy for fetal anomaly is regulated, in comparison to euthanasia cases. The latter is regulated by statute, with an exemption from liability in the Penal Code similar to art 296(5) WvS, the former through only Ministerial Regulations without an exemption from liability built into the Penal Code. The LTP Evaluations recommended that the Penal Code should be amended to include an exemption for termination after viability on the basis of an embryopathic (encompassing both categories) and a maternal indication: LTP Evaluation 2013 (n 89) 13, 106; LTP Evaluation 2022 (n 77) recommendation 22.

111 LTP Evaluation 2013 (n 89) 65–66, 106, 108; LTP Evaluation 2022 (n 77) 7.4.2, recommendation 20.

112 Written answer to parliamentary questions about going abroad for abortion by Minister De Jonge, Minister for Health, Wellbeing and Sport, 27/09/2021: <https://www.rijksoverheid.nl/binaries/rijksoverheid/documenten/kamerstukken/2021/09/27/beantwoording-kamervragen-over-het-artikel-zij-moest-naar-belgie-voor-de-abortus-van-haar-ernstig-gehandicapte-kind-omdat-artsen-verzwegen-dat-het-ook-in-nederland-kon/beantwoording-kamervragen-over-het-artikel-zij-moest-naar-belgie-voor-de-abortus-van-haar-ernstig-gehandicapte-kind-omdat-artsen-verzwegen-dat-het-ook-in-nederland-kon.pdf> accessed 16 March 2023.

113 Second Review of the Wafz (n 10) [3.5]; LTP Evaluation 2022 (n 77) 3.5.1, recommendation 1.

114 Second Review of the Wafz (n 10) 46, 60; LTP Evaluation 2013 (n 89) 12, 74, LTP Evaluation 2022 (n 77) 9ff, 66ff.

115 The 2022 LTP Evaluation acknowledged that the viability threshold results in pressure to make a decision before the 24th week. Putative parents interviewed reported that by opting for termination before that time they had a choice, albeit not necessarily a fully informed choice; a choice that might not be available to them after the viability threshold was met due to the narrow interpretation of the due care criteria, LTP Evaluation 2022 (n 77) 3.4.1.

116 For details of the reported impact upon pregnant persons see LTP Evaluation 2022 (n 77) 45–47, 67.

117 (n 87).

118 art 40 WvS.

119 Dutch Association for Obstetrics and Gynaecology (NVOG), *Modelprotocol: Medisch handelen bij beëindigen van de zwangerschap op maternale indicatie* (Model protocol: Medical treatment in the case of termination of pregnancy on maternal indication), 2017, [2.2].

120 Duijst *Tekst &Commentaar Gezondheidsrecht, commentaar op aanhef Wafz*, art 296 WvS.

121 The prosecution policy concludes that the doctor’s actions will be justified by necessity and that criminal investigation will not be indicated, Prosecution Decision (n 99) 5. However, the NVOG protocol requires all cases of late termination on a maternal indication to be reported to the Netherlands Obstetric Surveillance System to enable late pregnancy termination on maternal indication to be registered and analysed, NVOG model protocol (n 119) [3.2].

122 See (n 119).

123 Where such a threat exists, there is no question of waiting for the fetus to die before intervening, as has been reported in recent Polish cases: <https://www.theguardian.com/global-development/2022/jan/27/protests-flare-across-poland-after-death-of-young-mother-denied-an-abortion> accessed 17 March 2023.

124 NVOG model protocol (n 119) [2.2].

125 S Sheldon, ‘The Decriminalisation of Abortion: An Argument for Modernisation’ (2016) 36 Oxford Journal of Legal Studies 334; Erdman (n 1); S Halliday ‘Maintaining the Criminal Prohibition of Abortion as a Means of Protecting Women: Alternative Facts and Realities in reproductive Law and Policy’, in A Sinn and others (eds), *Populismus und alternative Fakten* (Mohr Siebeck 2020) 105; Romanis (n 4).

126 On the rhetoric and use of the criminal law to regulate abortion see Halliday (n 125).

127 AP Simester and A von Hirsch, *Crimes, Harms, and Wrongs: On the Principles of Criminalisation* (Hart 2011) 5. On the decriminalisation of abortion see Sheldon (n 14); S Sheldon and K Wellings (eds.), *Decriminalising Abortion in the UK: What Would It Mean?* (BUP 2019).

128 Sheldon (n 14) 344.

129 Department for Health and Social Care, ‘Abortion Statistics, England and Wales: 2021’ <https://www.gov.uk/government/statistics/abortion-statistics-for-england-and-wales-2021/abortion-statistics-england-and-wales-2021> accessed 24 March 2023. NB: we use the language of women here as this is what is used by the report.

130 Halliday (n 81); Halliday (n 125); EC Romanis and others, ‘The Excessive Regulation of early Abortion Medication in the United Kingdom: The Case for Reform’ (2022) 1 Medical Law Review 4; Parsons and Romanis (n 27).

131 M Heath and E Mulligan, ‘Abortion in the Shadow of the Criminal Law? The Case of South Australia’ (2016) 37 Adelaide Law Review 41, 65.

132 See also RJ Cook, ‘Stigmatized Meanings of Criminal Abortion Law’ in RJ Cook and others (eds), *Abortion law in Transnational Perspective: Cases and Controversies* (University of Pennsylvania Press 2014) 353; S Sheldon and others, ‘The Abortion Act 1967: A Biography’ (2019) 39 Legal Studies 18.

133 See, for example, BMA (n 25) [2.1.1], reporting doctors concerns about the risk of prosecution in relation to the exercise of their clinical judgement in the context of embryopathic abortion. See also S Saraiya, ‘Conceiving Criminality: An Evaluation of Abortion Decriminalization Reform in New York and Great Britain’ (2018) 57 Columbia Journal of Transnational Law 174. In *A, B and C v Ireland* [2010] ECHR 2032, the European Court of Human Rights recognised that ‘the criminal provisions of the 1861 Act would constitute a significant chilling factor for both women and doctors in the medical consultation process, regardless of whether or not prosecutions have in fact been pursued under that Act’, [254]. This case concerned three people who had travelled to England from Ireland in order to terminate their pregnancy and considered the application of the OAPA 1861 in Ireland, prior to the repeal of Article 40.3.3 Irish Constitution, at a time when abortion could only lawfully be performed in Ireland for the purpose of saving the pregnant person’s life, *AG v X* [1992] IESC 1. However, we suggest that the Court’s recognition of the impact of the criminal law was not limited to the Irish context, but applies more generally to the criminalisation of abortion in any jurisdiction. The chilling effect of the fact that abortion after 24 weeks constitutes a (potentially justifiable) crime notwithstanding a maternal or embryopathic indication in the Netherlands, is a recurrent theme throughout the LTP Evaluation 2022 (n 77).

134 WHO (n 9) 22.

135 LTP Evaluation 2022 (n 77) 9–10, 66ff.

136 ibid.

137 See also LTP Evaluation 2022 (n 77) 124.

138 LTP Evaluation 2022 (n 77) 13, 106.

139 art 2 Ministerial Regulations (n 87) imposes a mandatory referral to the Prosecution Authorities of all category 2 (non-fatal embryopathic) cases, even in cases where the Committee finds that the doctor has acted in accordance with the due care criteria.

140 See generally LTP Evaluation 2022 (n 77), particularly chapter 4.

141 Cf art 6 Ministerial regulations (n 87).

142
*R (Crowter) v Secretary of State for Health and Social Care* [2022] EWCA Civ 1559 [98] per Underhill LJ.

143 BMA (n 25) [2.1.1].

144 Second Review of the Wafz (n 10) 3.5.4; LTP Evaluation 2022 (n 77) 3.4.1. The Evaluation found that 43% of respondents reported that there is often ‘insufficient time to complete diagnostics between the 20-week ultrasound and the 24^th^ week of pregnancy’ and 21% reported that the 24th week threshold often causes pregnant people to ‘experience time pressure when making an informed choice about whether or not to continue the pregnancy if a serious abnormality is found,’ table 3.1. In the context of England and Wales similar concerns about time pressure have been expressed by the BMA, BMA (n 25) [2.1.1].

145 Tongue (n 47) 183; LTP Evaluation 2022 (n 77) 3.5.4.

146 See Halliday (n 125).

147 Eg, ‘Oxford mum denies trying to ‘procure miscarriage’ in 2021’ <https://www.oxfordmail.co.uk/news/20281951.oxford-mum-denies-trying-procure-miscarriage-2021/> accessed 17 March 2023.

148
*R v Sarah Louise Catt* (2012), unreported, 17 September 2012; Halliday (n 125) 119–20. Not all cases of suspected abortion will be prosecuted. In 2022, the Observer reported that people who have had a miscarriage, or stillbirth, are being subjected to police investigations, accused of having illegal abortions, <https://www.theguardian.com/world/2022/jul/02/women-accused-of-abortions-in-england-and-wales-after-miscarriages-and-stillbirths> accessed 17 March 2023.

149 See Halliday (n 125).

150 Cook (n 132), 348.

151 WHO (n 9) 22; W Nowicka, ‘Sexual and Reproductive Rights and the Human Rights Agenda: Controversial and Contested’ (2011) 19 Reproductive Health Matters 119; F de Londras and others, ‘The Impact of Criminalisation on Abortion-related Outcomes: A Synthesis of Legal and Health Evidence’ (2022) 7 BMJ Global Health e010409, doi:10.1136/bmjgh-2022-010409.

152 WHO (n 9) 24.

153 de Londras (n 151) 2.

154 Special Rapporteur on the right of everyone to the enjoyment of the highest attainable standard of physical and mental health, Interim Report to the General Assembly (2011) (UN Doc A/66/254); Special Rapporteur on torture and other cruel, inhuman or degrading treatment or punishment, Report to the Human Rights Council (2016) (UN Doc A/HRC/31/57).

155 art 16 (1) (e).

156 CEDAW (2018) Report of the inquiry concerning the United Kingdom of Great Britain and Northern Ireland under art 8 of the Optional Protocol to the Convention on the Elimination of All Forms of Discrimination against Women. CEDAW/C/OP.8/GBR/1.

157 The Abortion Act 1967 does not extend to Northern Ireland, instead abortion was regulated in Northern Ireland by the OAPA 1861. ss 58 and 59 OAPA were repealed in Northern Ireland by s 9 Northern Ireland (Executive Formation etc) Act 2019.

158 L Taylor, ‘How South America became a Global Role Model for Abortion Rights’ (2022) BMJ <https://doi.org/10.1136/bmj.o1908>.

159 EC Romanis, ‘The End of (Reproductive) Liberty as we know it: A Note on Dobbs V. Jackson Women’s Health 597 USC __ (2022),’ (2023) Medical Law International <https://doi.org/10.1177/096853322311545>.

160 J Herring and others, ‘Would Decriminalisation Mean Deregulation?’ in S Sheldon and K Wellings (eds), *Decriminalising Abortion in the UK* (Policy Press 2020) 58.

161 WHO (n 9) 26.

162 ibid 28.

163 C Borgmann, ‘Abortion exceptionalism and Undue Burden Preemption’ (2014) 71 Washington and Lee Law Review 1047; specifically in the English and Welsh context: Parsons and Romanis (n 27), and in the context of England and Germany: Halliday (n 81) and (n 125).

164 Sheldon (n 14) 346; Romanis and others (n 130) 18.

165 AM Sorhaindo and AF Lavelanet ‘Why does Abortion Stigma Matter? A Scoping Review and Hybrid Analysis of Qualitative Evidence Illustrating the Role of Stigma in the Quality of Abortion Care’ (2022) 311 Social Science & Medicine <https://doi.org/10.1016/j.socscimed.2022.115271>, 2.

166 Halliday (n 81); Halliday (n 125); Romanis and others (n 130), 9–18.

167 Sorhaindo (n 165), 2; WHO (n 9) 22, 61.

168 Telemedical abortion has reduced access barriers significantly in the UK (Parsons and Romanis (n 27)), but is not available in the Netherlands.

169 S Sheldon, *Beyond Control: Medical Power and Abortion Law* (Pluto Press 1997) 35.

170 If the abortion is immediately necessary a second signature is not required, s 1(4) AA 1967.

171 Halliday (n 125) 113.

172 The study concerned pregnancies over 20 weeks GA, including some individuals seeking abortion both before and after viability.

173 BPAS, *Why Women Present for Abortions After 20 Weeks* (2017) 8.

174 Romanis (n 4) 26.

175
*Re MB* [1997] EWCA Civ 309, [60]; J. de Boer and others, *Mr. C. Assers Handleiding tot de beoefening van het Nederlands Burgerlijk Recht. 1. Personen- en familierecht. Deel I. De persoon, afstamming en adoptie, gezag en omgang, levensonderhoud, bescherming van meerderjarigen*, Wolters Kluwer, 19th edn 2020, s 1 [34–35].

176 The significant differences between a fetus and a newborn are thoroughly explored for their moral and legal significance elsewhere, see: K Greasley, *Arguments about Abortion: Personhood, Morality and Law* (OUP 2017); Romanis (n 4).

177 Tongue (n 47) 185.

178 WHO (n 9) 28.

179 See (n 71).

180
*C v S* [1988] 1 QB 135, 151.

181 De Proost (n 4) 116.

182 Romanis (n 4) 3; LM Di Stefano and others, ‘Viability and Thresholds for Treatment of Extremely Preterm Infants: Survey of UK Neonatal Professionals’ (2021) 106 Archives of Disease in Childhood. Fetal and Neonatal Edition F596; R Wertheimer, ‘Understanding the Abortion Argument’ (1971) 1 Philosophy & Public Affairs 67, 82; L Han and others, ‘Blurred Lines: Disentangling the Concept of Fetal Viability from Abortion Law’ (2018) 28 Women’s Health Issues 287.

183 Di Stefano (n 182); HC Glass and others, ‘Outcomes for Extremely Premature Infants’ (2015) 120 Anesth Analg 1337; S Santhakumaran and others, ‘Survival of very Preterm Infants Admitted to Neonatal Care in England 2008-2014: Time Trends and Regional Variation’ (2018) 103 Archives of Disease in Childhood. Fetal and Neonatal Edition F208.

184 Second Review of the Wafz (n 10) 56 and 64.

185 Han (n 182) 287.

186 Romanis (n 4) 115.

187 K. Greasley (n 176) 190–91.

188 S Halliday, *Autonomy and Pregnancy: A Comparative Analysis of Compelled Obstetric Intervention* (Routledge 2016) 175–84.

189
*R v Sarah Louise Catt* (n 148).

190 L Eades, ‘Social Realities, Biological Realities: The 24-week Foetus in Contemporary T English Abortion Activism’ (2019) 74 Women's Studies International Forum 20, 21.

191 See also de Londras (n 151) 4, 6.

